# The foliar potassium silicate enhances wheat terminal-drought tolerance and yield via physiological, biochemical and genetic regulation

**DOI:** 10.3389/fpls.2026.1766913

**Published:** 2026-05-04

**Authors:** Yahya Alzahrani, Hameed Alsamadany, Zahid H. Shah

**Affiliations:** 1 Department of Biological Sciences, Faculty of Science, King Abdulaziz University, Jeddah, Saudi Arabia; 2 College of Agriculture, Jilin Agricultural University, Changchun, China

**Keywords:** antioxidant, biochemical, gene regulation, gene-trait association, physiological, water relations

## Abstract

Drought stress limits wheat productivity at terminal stage by disrupting physiological activities, impairing cellular homeostasis, and decreasing grain yield. Present study elucidated the role of potassium silicate (K_2_SiO_3_) in mitigating drought-induced damage in wheat through integrated physiological, biochemical, agronomic, and molecular approaches. Wheat plants were subjected to terminal drought-stress under four different levels (0, 2, 4, and 6 mM) of K_2_SiO_3_. Application of K_2_SiO_3_, particularly at 6 mM, significantly improved relative water content, photosynthetic rate, and PSII efficiency. Antioxidant defense system was reinforced through higher SOD, CAT, and POD activities, accompanied by lower MDA and electrolyte leakage. Yield components were markedly increased, with grain yield increasing by 72% relative to control, alongside significant gains in thousand grain weight, grains per spike, and biomass. Gene expression analysis revealed significant upregulation of *TaDREB2* and *TaNCED1*, supporting improved water relations, while *TaSOD, TaCAT, and TaAPX1* regulation corresponded with increased antioxidant enzymes activities. Reduced expression of *TaP5CS* aligned with lower proline accumulation, whereas *TaBADH* upregulation was consistent with increased GB content. Moreover, *TaLEA* upregulation supported membrane stability, and yield-related genes *TaCKX2* and *TaGW2* expression improved spike fertility and grain weight. These results demonstrated that K_2_SiO_3_ treatment activates multiple drought tolerance mechanisms that integrate gene regulation, osmotic adjustment, antioxidant defense, and yield stabilization. Overall, the findings establish application of K_2_SiO_3_ especially at 6 mM, as an effective strategy for enhancing drought tolerance and sustaining the productivity of drought susceptible wheat cultivar (Galaxy-2013) at terminal stage of drought stress.

## 1 Introduction

Wheat (*Triticum aestivum* L.) is the globally cultivated cereal crop, contributing nearly 20% of dietary calories and protein intake in the world (FAO, 2025). However, its productivity is highly vulnerable to water stress, especially in arid and semi-arid regions where drought during terminal reproductive stages reduces photosynthetic, accelerates leaf senescence, disrupts nutrient assimilation, and reduces grain number and weight (Farooq et al., 2014; Mwadzingeni et al., 2016). The climate change is exacerbating the frequency and severity of drought, therefore development of effective agronomic strategies to enhance wheat tolerance has become a major challenge for global food security (Jangra et al., 2019; Zhang et al., 2022).

Potassium silicate (K_2_SiO_3_) is widely recognized as an effective source of both potassium (K) and silicon (Si), which play important roles in enhancing plant growth and improving tolerance to environmental stresses (Coskun et al., 2019; Wang et al., 2013). Potassium is a vital macronutrient involved in several physiological processes, including enzyme activation, osmotic adjustment, regulation of stomatal movement, and maintenance of cellular homeostasis, thereby contributing to improved water-use efficiency and plant productivity under stress conditions (Nomenaharinaivo et al., 2025). Silicon (Si), although not considered an essential nutrient for plants, is widely seen as helpful, especially in protecting plants from stresses such as drought, salinity, and heavy metal toxicity (Liang et al., 2015). In cereals, Si deposits in epidermal tissues, where it decreases non-stomatal water loss and strengthens structural integrity. Additionally, Si improves photosynthetic efficiency, osmotic adjustment, and antioxidant defense through limiting oxidative damage under stress (Gong et al., 2005; Coskun et al., 2019; Ashfaq et al., 2025). Different studies explained that Si application can affect gene expression, including upregulation of antioxidant genes (*TaSOD*, *TaCAT*, *TaAPX*), osmolyte biosynthesis genes (*TaP5CS*, *TaBADH*), and stress-related transcription factors (*TaDREB*, *TaNAC*), therefore combining physiological benefits with molecular mechanisms (Jiang et al., 2019; Wang et al., 2021; Li et al., 2023).

Among available Si sources, potassium silicate (K_2_SiO_3_) is more effective for foliar application due to its high solubility, rapid leaf absorption, and provision of both potassium and silicon (Pei et al., 2010; Johnson et al., 2022). In wheat, foliar application of K_2_SiO_3_ at concentrations between 2–6 mM has been reported to increase relative water content (RWC), chlorophyll, antioxidant enzyme activity, and yield-related traits under drought conditions (Gong et al., 2003, Gong et al., 2005; Aurangzaib et al., 2022; Johnson et al., 2022). Moreover, K_2_SiO_3_ activates the antioxidant defense genes such as *TaSOD*, *TaCAT*, and *TaAPX*, which detoxify the reactive oxygen species (ROS), and osmoprotectant genes such as *TaP5CS* and *TaBADH*, which regulate proline and glycine betaine (GB) accumulation for osmotic adjustment (Zhao et al., 2023; Rao et al., 2025). Besides, the regulation of key transcription factors further strengthens this protective mechanism. For instance, *TaDREB* and *TaNAC* genes regulate the drought-responsive pathways and increase stress signaling, while ABA biosynthesis gene *TaNCED1* contributes to stomatal conductance and water retention (Song et al., 2024). At the yield level, genes such as *TaCKX2* (cytokinin oxidase/dehydrogenase) and *TaGW2* (grain size regulator) have been associated with improved grain filling and spike traits under stress conditions (Zhang et al., 2018; Jablonski et al., 2021). Recent studies highlighted that enhanced foliar uptake of Si, increases its stress-mitigation effects by modulating physiological and biochemical mechanisms through regulating the drought-associated genes (Ning et al., 2023; Abdelaziz et al., 2024). Although past studies explained the beneficial effects of K_2_SiO_3_ on wheat growth and yield, most have focused on general conditions or early-stage stress. However, limited knowledge is available on the role of foliar-applied K_2_SiO_3_ under terminal-drought stress, particularly in context of its combined effects on physiological, biochemical, and genetic responses. Terminal drought is a main constraint that severely impacts wheat productivity at the reproductive stage, yet the underlying mechanisms of silicon-mediated tolerance at this stage remained least explored. Therefore, the present study was designed to elucidate how foliar K_2_SiO_3_ enhances wheat tolerance to terminal drought by regulating key physiological traits, biochemical processes, and gene expression, ultimately improving yield. Therefore, the present study aimed to evaluate the effects of foliar-applied K_2_SiO_3_ at three concentrations (2, 4, and 6 mM) on drought tolerance in wheat under controlled pot conditions. The objective was to identify the most effective concentration for improving water relations, physiological processes, antioxidant defense, gene expression, and yield components under terminal drought, thereby contributing to the development of silicon-based foliar strategies for wheat drought tolerance in water-limited environments.

## 2 Materials and methods

### 2.1 Plant material and growth medium

A single, high-yielding and drought susceptible bread wheat cultivar ‘Galaxy-2013’ (Ahmad et al., 2022) was used in this study. Certified seeds were obtained from an accredited national source (NARC, Islamabad, Pakistan) to ensure genetic purity. Seeds were surface-sterilized using 2% sodium hypochlorite for 3 minutes to remove seed-borne pathogens, followed by thorough rinsing in distilled water and air-drying at room temperature before sowing. The experiment was performed in plastic pots (30 cm diameter × 35 cm depth), each filled with 10 kg of a homogenized growth medium composed of loamy soil, river sand, and well-decomposed farmyard manure in a 2:1:1 **(v/v/v)**. The soil used had pH 7.5, EC1.8 dS m^-1^, and organic matter 0.9%. Field capacity and permanent wilting point were estimated gravimetrically to facilitate the drought stress management.

### 2.2 Experimental design and treatments

The experiment was carried out in a Randomized Complete Block Design (RCBD) with four potassium silicate (K_2_SiO_3_) treatments and three replicates. The experiment included four concentrations of potassium silicate (0, 2, 4, and 6 mM), where 0 mM served as the control. All treatments were subjected to terminal drought stress to evaluate the effectiveness of K_2_SiO_3_ in improving drought tolerance. Treatments were applied as T1-drought stress without K_2_SiO_3_, T2-drought + K_2_SiO_3_ at 2 mM (Pei et al., 2010), T3- drought + K_2_SiO_3_ at 4 mM (Bybordi, 2015), and T4- drought + K_2_SiO_3_ at 6 mM (Gong et al., 2003). Each pot was sown with eight seeds, later thinned to five uniform plants after emergence. Plants were grown under natural light in a greenhouse with day/night temperatures of 25–30 °C/15–20 °C and relative humidity of 55–65%. Terminal drought stress was applied at anthesis (Feekes growth stage 10.5) until early grain filling (Feekes growth stage 11.1), by restricting water application to maintain ~40% field capacity (Blum, 2011). The span of stress period was 25 days. Field capacity (FC) at 40% was calculated by gravimetric method. First, field capacity at 100% was measured by fully saturating the soil. Afterward the soil was freely drained, and then oven-dried at 105 °C for 24 h to determine dry weight. The required moisture levels were maintained by regularly weighing the pots. Soil moisture level corresponding to 40% of field capacity was measured as 40% of the water content at 100% field capacity and were maintained throughout the experimental period. Following equation was used for measuring the field capacity.

FC=(Wf-Wd/Wd) x 100, whereas Wf = fresh soil weight and Wd = oven-dry soil weight.

Soil moisture levels were monitored daily using a tensiometer and gravimetric soil moisture method. Besides, a non-stressed control was kept under identical K_2_SiO_3_ foliar application treatments, ensuring that any observed differences in growth, yield, or physiological parameters can be attributed specifically to drought-mitigation effects rather than general growth promotion. Each pot containing five plants was treated as an experimental unit since treatments were applied at the pot level. Five pots per treatment were maintained within each replicate. Measurements were recorded from randomly selected plants within each pot and averaged before statistical analysis. Because a potassium-only control was not included, the present study demonstrates the effect of potassium silicate (K_2_SiO_3_) as a treatment rather than isolating silicon-specific mechanisms.

### 2.3 Foliar potassium silicate application

K_2_SiO_3_ (Thermo Fisher Scientific, USA) of analytical grade (≥99% purity, SiO_2_ content 25–28%) was used as the silicon source. Moreover, the required concentrations were prepared by dissolving K_2_SiO_3_ in distilled water and adjusting the solution pH to 6.0–6.5 with dilute HCl to stabilize the silicon solubility (Liang et al., 2015). A non-ionic surfactant (Tween-20 at 0.05–0.1%) was added to enhance leaf surface wetting. Foliar sprays were applied twice, first at anthesis and the second 7–10 days later at early grain filling, using a fine-mist handheld sprayer calibrated to supply 300–500 L ha^-1^ equivalent volume. Foliar treatments were applied in the early morning or late afternoon to minimize evaporation losses and avoid leaf scorching (Ma and Yamaji, 2006). Leaves were sprayed till both adaxial and abaxial surfaces were wetted.

### 2.4 Physiological measurements

Physiological traits were measured at mid-anthesis. Relative water content (RWC) percentage was determined opting the standard procedure (Barrs and Weatherley, 1962). Besides, SPAD chlorophyll index was calculated with a SPAD-502 meter (Konica Minolta, Japan). Net photosynthetic rate (Pn) and stomatal conductance (Gs) were measured using a portable IRGA (infrared gas analyzer) (LI-6400XT, LI-COR Biosciences, USA). Midday leaf temperature (LT) was recorded by an infrared thermometer, and leaf water potential (Ψ_leaf) was calculated using a Scholander-type pressure chamber (Scholander et al., 1965). Maximum quantum efficiency of PSII (Fv/Fm) was calculated with the help of a portable pulse-amplitude-modulated fluorometer (PAM-2100, Walz, Germany).

### 2.5 Biochemical analyses

Flag leaves from wheat were sampled at mid-anthesis for biochemical assays. Proline content (Pro) was estimated using the acid ninhydrin method (Bates et al., 1973) and glycine betaine (GB) was estimated using the periodide assay (Grieve and Grattan, 1983). Antioxidant enzyme activities such as superoxide dismutase (SOD), catalase (CAT), and peroxidase (POD) were recorded spectrophotometrically using the protocols of Giannopolitis and Ries (1977); Aebi (1984), and Chance and Maehly (1955), respectively. Lipid peroxidation was estimated as malondialdehyde (MDA) content by the TBARS assay (Heath and Packer, 1968). Membrane stability was assessed in the form electrolyte leakage (EL) following the procedure used by Lutts et al. (1996).

### 2.6 Phenotypic and agronomic traits

At the state of physiological maturity, data were measured for plant height (PH), spike length (SL), grains per spike (GPS), tillers per plant (TPP), total above-ground biomass (BM), grain yield per plant (GY), thousand-grain weight (TGW), and harvest index (HI), calculated as the ratio of grain yield to total biomass.

### 2.7 Statistical analysis

The tri-replicated experiment was executed in randomized complete block design (RCBD). One way ANOVA was performed to assess the effects of different levels of K_2_SiO_3_ on the measured traits of drought susceptible wheat cultivar ‘Galaxy-2013’ under terminal drought stress using Statistix (version 8.1) at a 5% probability level. When ANOVA illustrated significant differences, mean values of traits were separated using the Least Significant Difference (LSD) test at p≤ 0.05. Moreover, the uncertainty around the estimated means was expressed by calculating 95% confidence intervals (CIs) for all measured traits. Additionally, effect sizes were computed where appropriate to indicate the magnitude of treatment effects. The assumptions of normality and homogeneity of variance were examined before performing the analysis.

Correlation and principal component analysis (PCA) were performed using mean values of physiological, biochemical and yield traits from all treatments to examine the relationships among measured variables under drought stress and potassium silicate treatments. Moreover, RStudio version 1.1.456 (RStudio Team, 2020) was used to construct correlation chart, heatmap dendrogram and principal component analysis (PCA) biplot. The R packages “GGally” and “ggplot2” were used to execute Pearson correlation, while the R packages “factoextra” and “FactoMineR” were used to establish the PCA biplot. PCA was performed on mean-centered and unit variance–scaled data, and components with eigenvalues >1 were retained. Besides, the heatmap was prepared using the R packages, “pheatmap” and “complex Heatmap”. using Euclidean distance for clustering and complete linkage as the aggregation method, with trait values standardized to z-scores prior to plotting.

### 2.8 Gene expression analysis

RNA was extracted from flag leaves using TRIzol, (Chomczynski and Sacchi, 1987) treated with DNase I, and reverse-transcribed into cDNA. Furthermore, Quantitative PCR was performed using SYBR Green in 10 µL reactions (Bustin et al., 2009) using primers (**Table 1**) for drought-related genes (*TaDREB2, TaNCED1, TaSOD, TaCAT, TaAPX1, TaP5CS, TaBADH, TaLEA, TaGW2*, and *TaCKX2*), with *TaActin* as reference. The amplification conditions included initial denaturation at 95 °C for 2–3 min, followed by 35–40 cycles of denaturation at 95 °C for 10–15 s, annealing at 55–60 °C for 20–30 s, and extension at 72 °C for 20–30 s. A melt curve analysis was performed to confirm amplification specificity, and all reactions were conducted in triplicate. Primer specificity and efficiencies (90–110%) were validated, and relative expression was calculated by the ΔΔCt method (Livak and Schmittgen, 2001). Relative gene expression was analyzed using the ΔΔCt method in qRT-PCR. Ct values of target genes were normalized with respect to reference gene to obtain ΔCt. Moreover, ΔΔCt was measured by comparing treated samples with the control. Relative expression levels were expressed as fold changes using the formula 2^−ΔΔCt. Data (mean ± SE) from three biological and three technical replicates were analyzed by ANOVA with Tukey’s test (p < 0.05).

**Table 1:** List of drought associated genes (with primer sequence, and trait associated) used in relative expression analysis.

Gene symbol	Primer sequence (5′–3′)	Trait associated	Reference
*TaDREB2*	F: AGGACTACGACGAGGAGGAA R: TTGCTTGTTGCTGTTGCTGT	Regulation of stress-inducible genes, RWC, Pn	Wang et al., 2021
*TaNCED1-5B*	F: CAGTGGCTGGAGGAAGAGTT R: AGGTGGTGATGTTGAGGAGG	ABA regulation, stomatal closure, drought tolerance	Song et al., 2024
*TaSOD*	F: ATGGCCAAACAGTTCCTTCC R: TTAGTCCACACCGACGACAC	Antioxidant defense, reduced oxidative stress	Jiang et al., 2019
*TaCAT*	F: GACGATGGAGACGTTGGTGA R: ACGTGGGTGAGTAGCAGGAT	Antioxidant defense, lower lipid peroxidation and membrane stability	Zhang et al., 2022
*TaAPX1*	F: CAGTTCGACCTCGATGAGGA R: TGATGGCGTAGTTCCTCCTT	ROS detoxification, reduced EL	Madhu et al., 2024
*TaP5CS*	F: TGTGGTGGTGGTGTTCTTGT R: AGTTGGTGGTGATGGTGTTG	Proline accumulation and osmotic protection	Aycan et al., 2022
*TaBADH-A1*	F: GAGAGGAGCTGGAGGTTGAG R: TGGAGGGTGTAGTGGAGGAG	GB accumulation and osmotic protection	Zhao et al., 2023
*TaLEA*	F: ATGCTCGCTCTCGCTCTC R: GCTTGGTGGTTGGTGGTAG	Protein and membrane stabilization	Ali et al., 2020
*TaGW2*	F: TGGTGTTGGTTGGTTGGTTG R: AGGTTGGTGGTGTTGGTGGT	TGW, yield	Zhang et al., 2018
*TaCKX2*	F: ATCGACCTTGGTGATGGTGT R: CGTCTTGGTGTAGTGGGTTG	GPS, yield	Abdelaziz et al., 2024

## 3 Results

### 3.1 Physiological responses to K_2_SiO_3_ under drought stress

**Figure 1:**
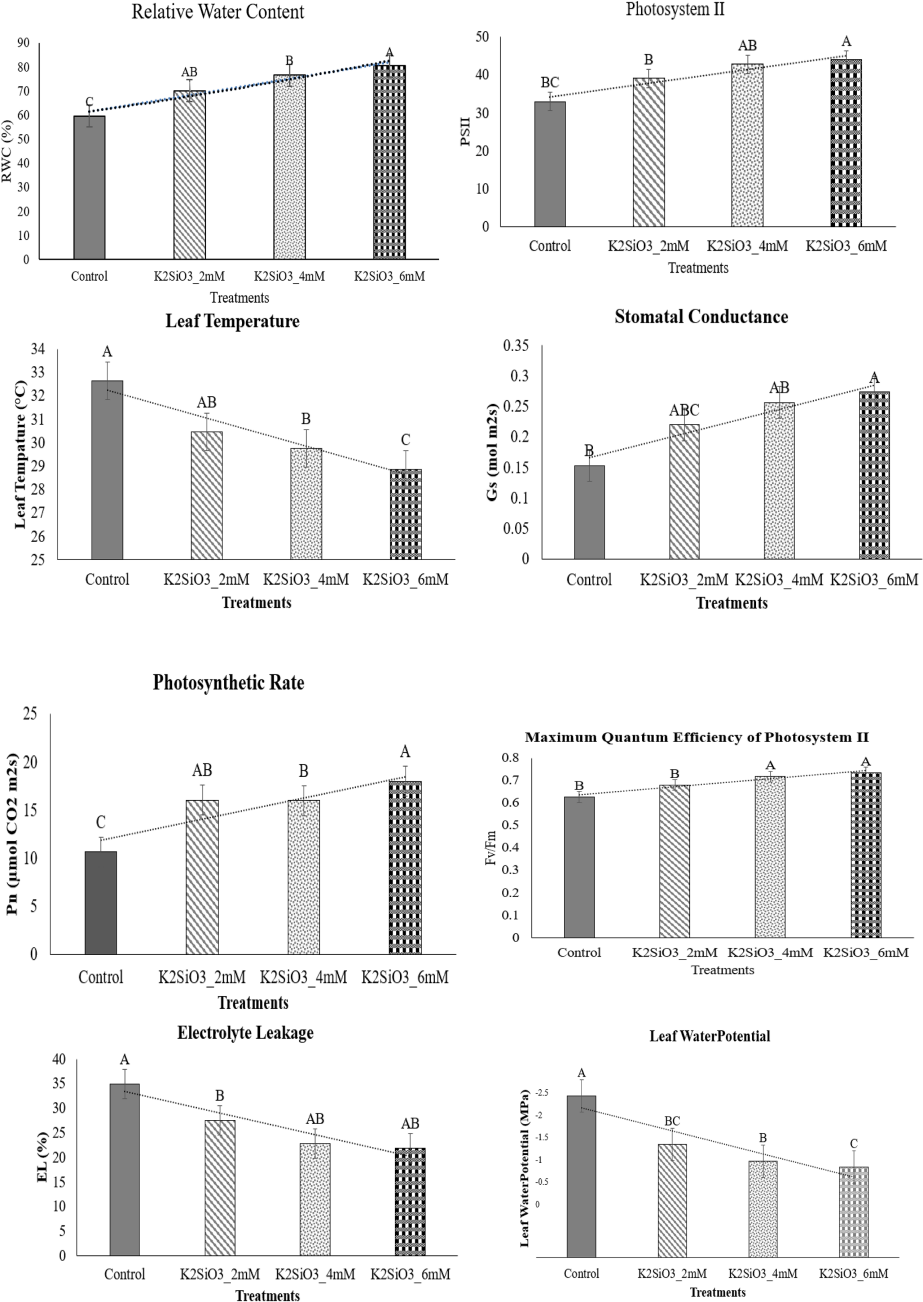
Effect of potassium silicate (K_2_SiO_3_) application on physiological attributes of the drought-susceptible wheat cultivar ‘Galaxy-2013’ under terminal drought stress. The evaluated parameters included relative water content (RWC), photosystem II efficiency (PSII), leaf temperature (LT), stomatal conductance (Gs), net photosynthetic rate (Pn), maximum quantum efficiency of PSII (Fv/Fm), electrolyte leakage (EL), and leaf water potential (LWP). Data are presented as mean ± standard error (SE) of three biological replicates (n = 3). Bars sharing different letters indicate statistically significant differences among treatments at *p* ≤ 0.05, as determined by the least significant difference (LSD) test.

Application of potassium silicate (K_2_SiO_3_) significantly (p ≤ 0.05) improved the physiological traits of wheat under terminal drought stress (**Figure 1**). Relative water content (RWC) increased from 62.4% in the control to 74.8% at 2 mM, 71.3% at 4 mM, and 79.6% at 6 mM K_2_SiO_3_, with the maximum improvement at 6 mM (**Figure 1**). Similarly, Photosystem II activity (PSII) increased significantly (p ≤ 0.05) from 30.2 in the control to 36.5, 34.8, and 42.1 under 2, 4, and 6 mM treatments, respectively (**Figure 1**). Leaf temperature decreased from 33.5 °C in the control to 31.4 °C, 29.6 °C, and 28.4 °C under 2, 4, and 6 mM treatments (**Figure 1**). Stomatal conductance (Gs) showed a significant increasing trend (p < 0.05), rising from 0.14 mol m^-2^ s^-1^ in the control to 0.19, 0.23, and 0.28 mol m^-2^ s^-1^ under 2, 4, and 6 mM treatments, respectively (**Figure 1**). Photosynthetic rate (Pn) also showed a significant increase, rising from 11.5 µmol CO_2_ m^-2^ s^-1^ in the control to 16.8, 18.7, and 21.3 µmol CO_2_ m^-2^ s^-1^ under 2, 4, and 6 mM treatments, respectively (**Figure 1**). Maximum quantum efficiency of PSII (Fv/Fm) increased from 0.68 in the control to 0.71, 0.72, and 0.75 in treated plants (**Figure 1**). Electrolyte leakage (EL), an indicator of membrane injury, was significantly (p < 0.05) reduced from 35.6% in control to 24.3%, 21.7%, and 19.6% at 2, 4, and 6 mM, respectively (**Figure 1**). Leaf water potential (LWP) also improved, increasing from –2.45 MPa in control to –1.93, –1.72, and –1.58 MPa in 2, 4, and 6 mM treatments. These findings highlight that K_2_SiO_3_ substantially mitigated water deficit stress, with the 6 mM treatment showing maximum physiological improvement.

### 3.2 Biochemical adjustments under K_2_SiO_3_ treatments

**Figure 2:**
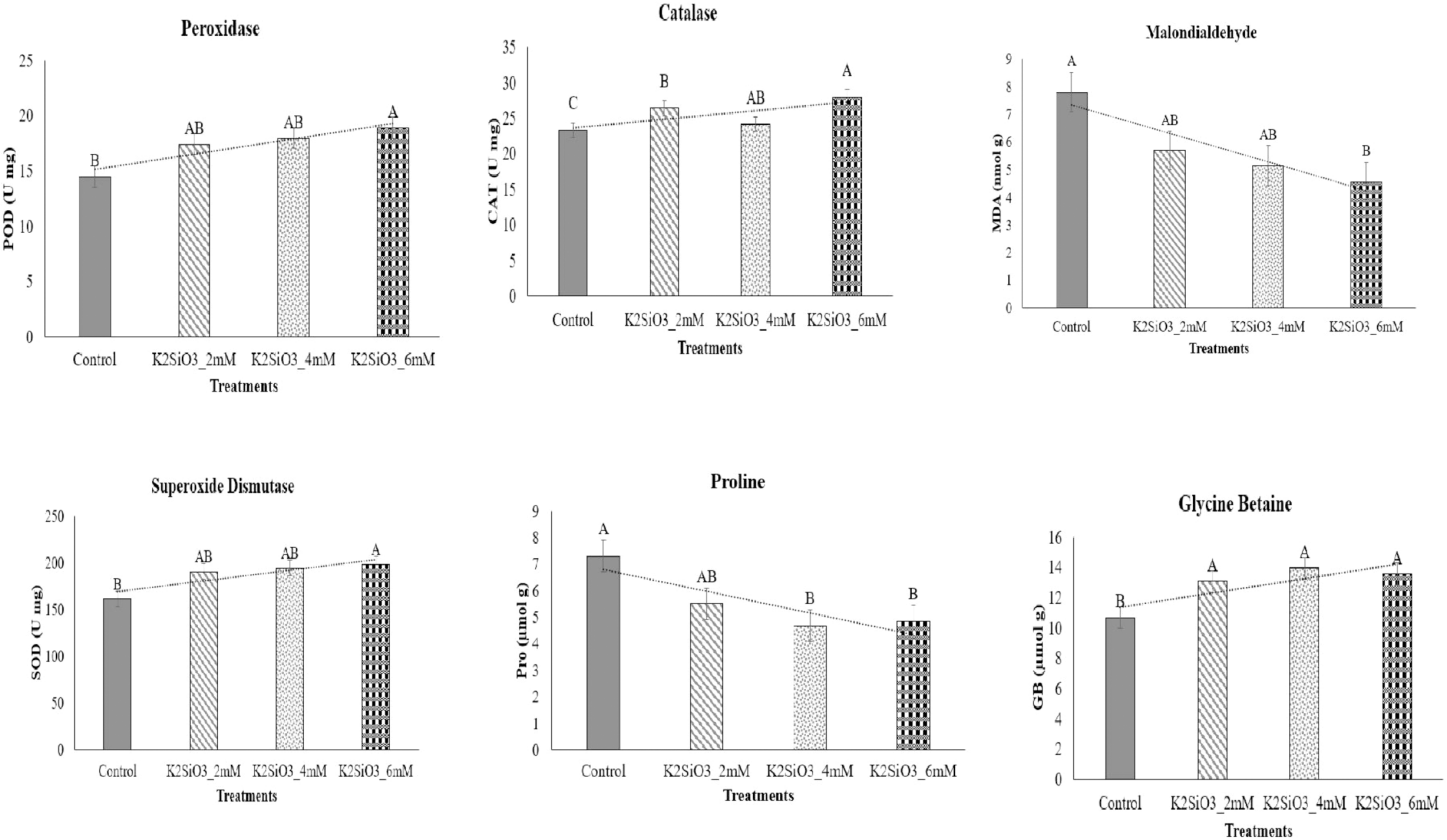
Effect of potassium silicate (K_2_SiO_3_) application on biochemical responses of the drought-susceptible wheat cultivar ‘Galaxy-2013’ under terminal drought stress. The assessed biochemical parameters included peroxidase (POD), catalase (CAT), malondialdehyde (MDA), superoxide dismutase (SOD), proline, and glycine betaine (GB). Data are presented as mean ± standard error (SE) from three biological replicates (n = 3). Bars with different letters denote statistically significant differences among treatments at *p* ≤ 0.05, as determined by the least significant difference (LSD) test.

Significant changes (p < 0.05) in antioxidant activity and osmolyte accumulation were observed under K_2_SiO_3_ treatments (**Figure 2**). Peroxidase (POD) activity increased from 14.2 U mg^-1^ protein in control to 17.6, 18.9, and 21.2 U mg^-1^ protein under 2, 4, and 6 mM K_2_SiO_3_, respectively (**Figure 2**). Catalase (CAT) activity was significantly (p < 0.05) enhanced from 22.4 U mg^-1^ protein in control to 26.8, 28.4, and 30.9 U mg^-1^ protein in 2, 4, and 6 mM treatments (**Figure 2**). Likewise, superoxide dismutase (SOD) activity increased significantly (p < 0.05) from 155 U mg^-1^ protein in control to 182, 188, and 203 U mg^-1^ protein at 2, 4, and 6 mM, respectively (**Figure 2**).

Malondialdehyde (MDA), a lipid peroxidation marker, decreased significantly (p < 0.05) from 8.4 nmol g^-1^ FW in the control to 6.9, 6.1, and 5.2 nmol g^-1^ FW under 2, 4, and 6 mM treatments, respectively (**Figure 2**). Proline content, which was higher in stressed control plants (7.6 µmol g^-1^ FW), decreased significantly (p < 0.05) with K_2_SiO_3_ application to 6.1, 5.2, and 4.8 µmol g^-1^ FW under 2, 4, and 6 mM treatments, respectively (**Figure 2**). In contrast, glycine betaine (GB) accumulated significantly (p < 0.05), increasing from 9.2 µmol g^-1^ FW in the control to 12.5, 13.8, and 14.6 µmol g^-1^ FW under 2, 4, and 6 mM treatments, respectively (**Figure 2**). Collectively, these biochemical responses demonstrated that K_2_SiO_3_ improved oxidative defense and osmolyte regulation under drought stress.

### 3.3 Yield-related traits under K_2_SiO_3_ application

**Figure 3:**
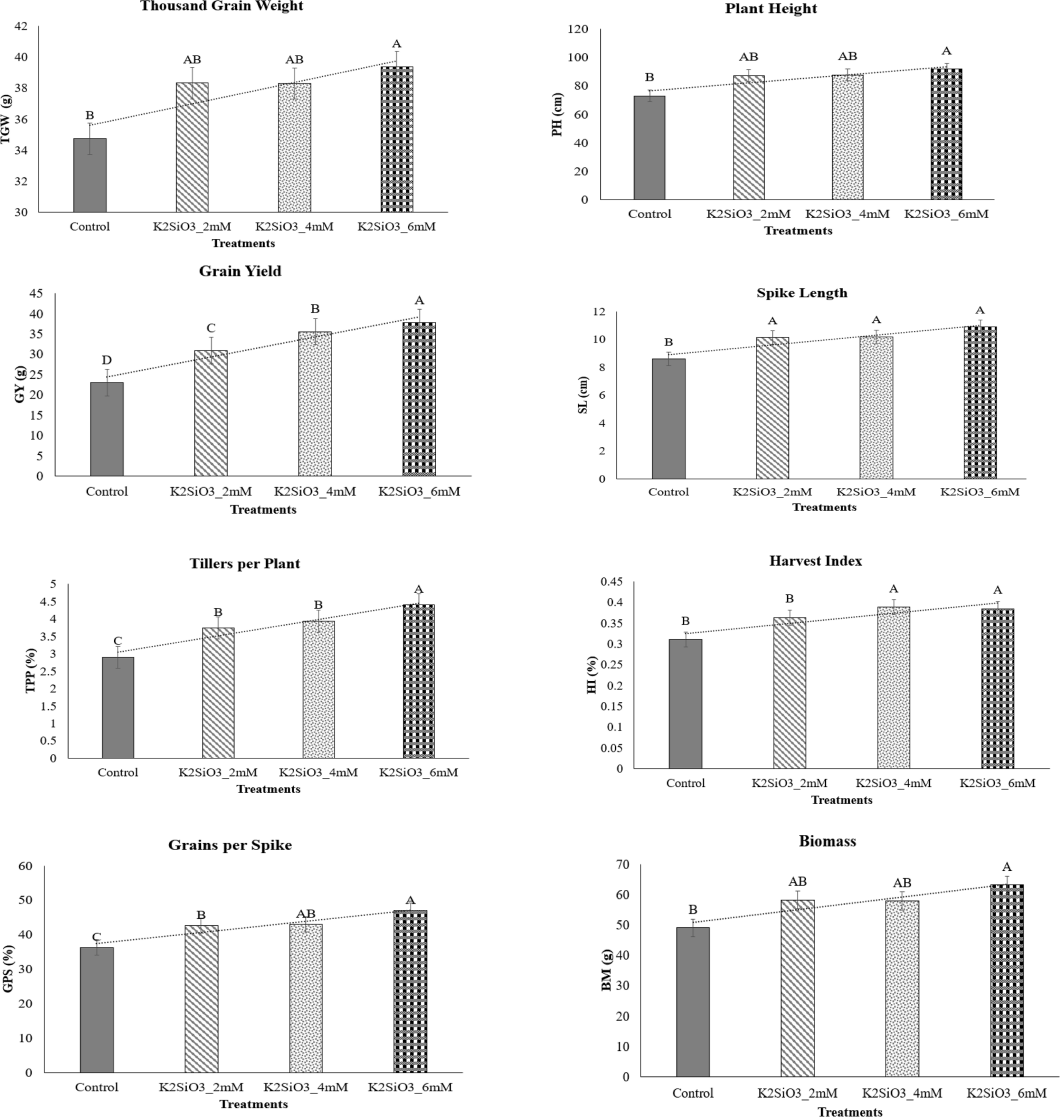
Effect of potassium silicate (K_2_SiO_3_) application on yield-related traits of the drought-susceptible wheat cultivar ‘Galaxy-2013’ under terminal drought stress. **T**he evaluated yield attributes included thousand grain weight (TGW), plant height (PH), grain yield (GY), spike length (SL), tillers per plant (TTP), harvest index (HI), grains per spike (GPS), and biomass (BM). Data are presented as mean ± standard error (SE) of three biological replicates (n = 3). Bars sharing different letters indicate statistically significant differences among treatments at *p* ≤ 0.05, based on the least significant difference (LSD) test.

Yield and agronomic traits showed marked improvement (p < 0.05) with K_2_SiO_3_ application (**Figure 3**). Thousand grain weight (TGW) increased from 33.1 g in control **to** 36.4, 37.5, and 39.8 g at 2, 4, and 6 mM, respectively (**Figure 3**). Plant height (PH) improved significantly (p < 0.05) from 75.3 cm in control to 84.6, 86.1, and 90.7 cm with treatments (**Figure 3**). Grain yield (GY) increased dramatically (p < 0.05) from 21.3 g plant^-1^ in control to 27.5, 32.4, and 36.7 g plant^-1^ under 2, 4, and 6 mM treatments, respectively, showing the highest significance at 6 mM (**Figure 3**). Spike length (SL) also improved significantly (p < 0.05), increasing from 8.4 cm in control to 9.2, 9.6, and 10.3 cm at 2, 4, and 6 mM concentrations of K_2_SiO_3_ (**Figure 3**). Tillers per plant (TTP) rose significantly (p < 0.05) from 2.7 in control to 3.4, 3.6, and 4.2, while harvest index (HI) improved from 0.28 in control to 0.33, 0.36, and 0.37 in respective treatments (**Figure 3**). Grains per spike (GPS) significantly (p < 0.05) increased from 38.5 in control to 43.2, 46.1, and 49.6 under treatments (**Figure 3**). Likewise, biomass (BM) accumulation improved significantly (p < 0.05) from 52.3 g plant^-1^ in control to 58.6, 61.4, and 65.8 g plant^-1^ under 2, 4, and 6 mM, respectively (**Figure 3**). Overall, yield components showed a treatment-dependent response, with 6 mM K_2_SiO_3_ producing maximum grain yield and biomass under drought stress.

### 3.4 Correlation, PCA biplot, ellipse and heatmap analysis

**Figure 4:**
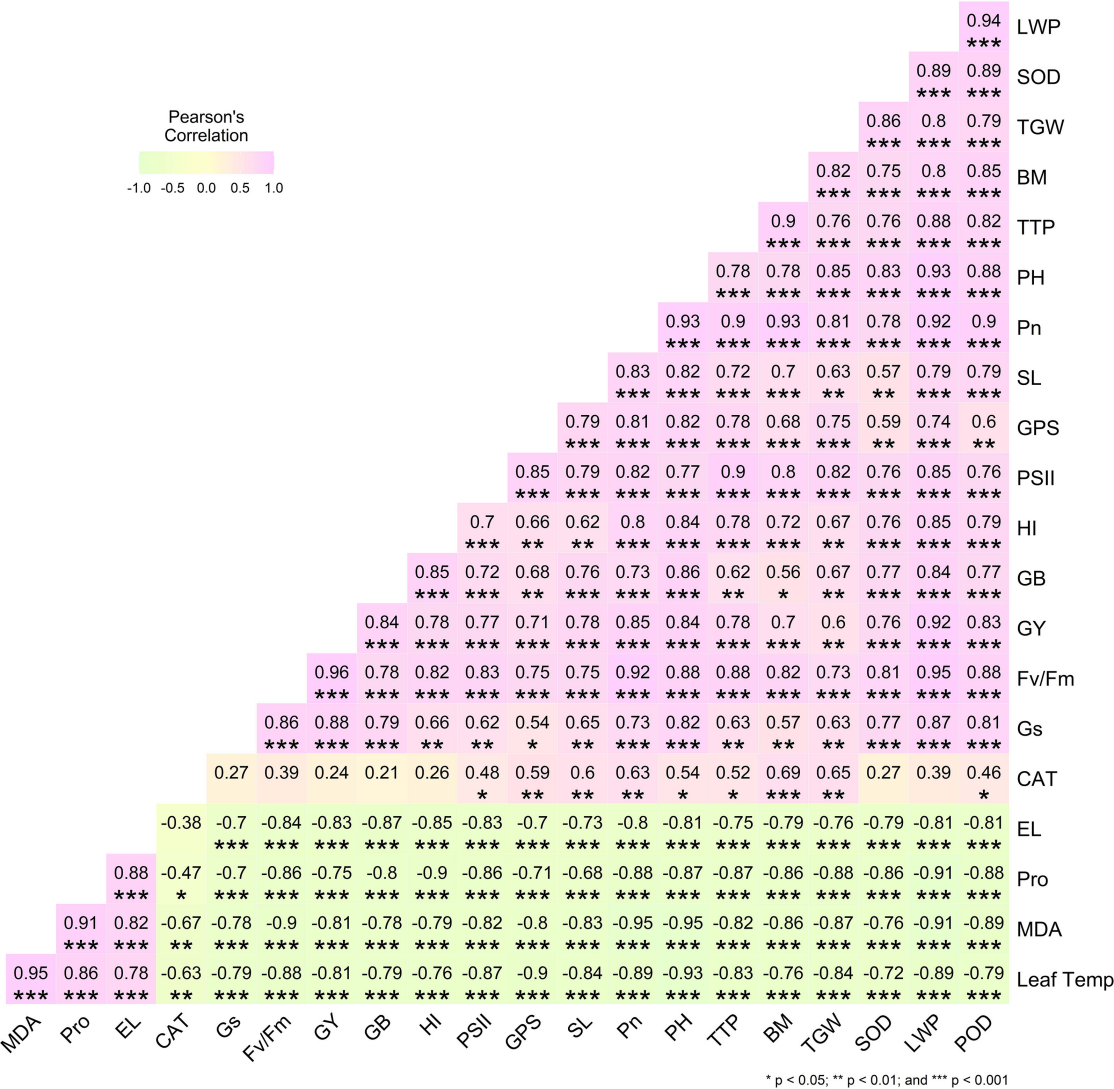
Pearson’s correlation matrix illustrating pairwise relationships among physiological, biochemical, and yield-related traits of the drought-susceptible wheat cultivar ‘Galaxy-2013’ under potassium silicate (K_2_SiO_3_) treatments during terminal drought stress. Positive correlations are shown in purple, whereas negative correlations are depicted in green. Color intensity and the magnitude of correlation coefficients reflect the strength of associations. Asterisks denote levels of statistical significance (*p* < 0.05, p < 0.01, *p* < 0.001).

Pearson’s correlation matrix depicted strong positive paired associations between yield traits (GY, TGW, GPS, PH, BM) and physiological parameters such as RWC (r = 0.96***), Pn (r = 0.93***), PSII (r = 0.85***), and Gs (r = 0.84***) as shown in **Figure 4**. Moreover, Fv/Fm was also strongly correlated with yield (r = 0.88***). Conversely, negative correlations were observed between stress indicators such as MDA (r = –0.95***), EL (r = –0.88***), proline (r = –0.91***), and leaf temperature (r = –0.79**) with yield traits, highlighting the protective role of K_2_SiO_3_ in reducing oxidative damage and improving performance under terminal drought stress (**Figure 4**).

**Figure 5:**
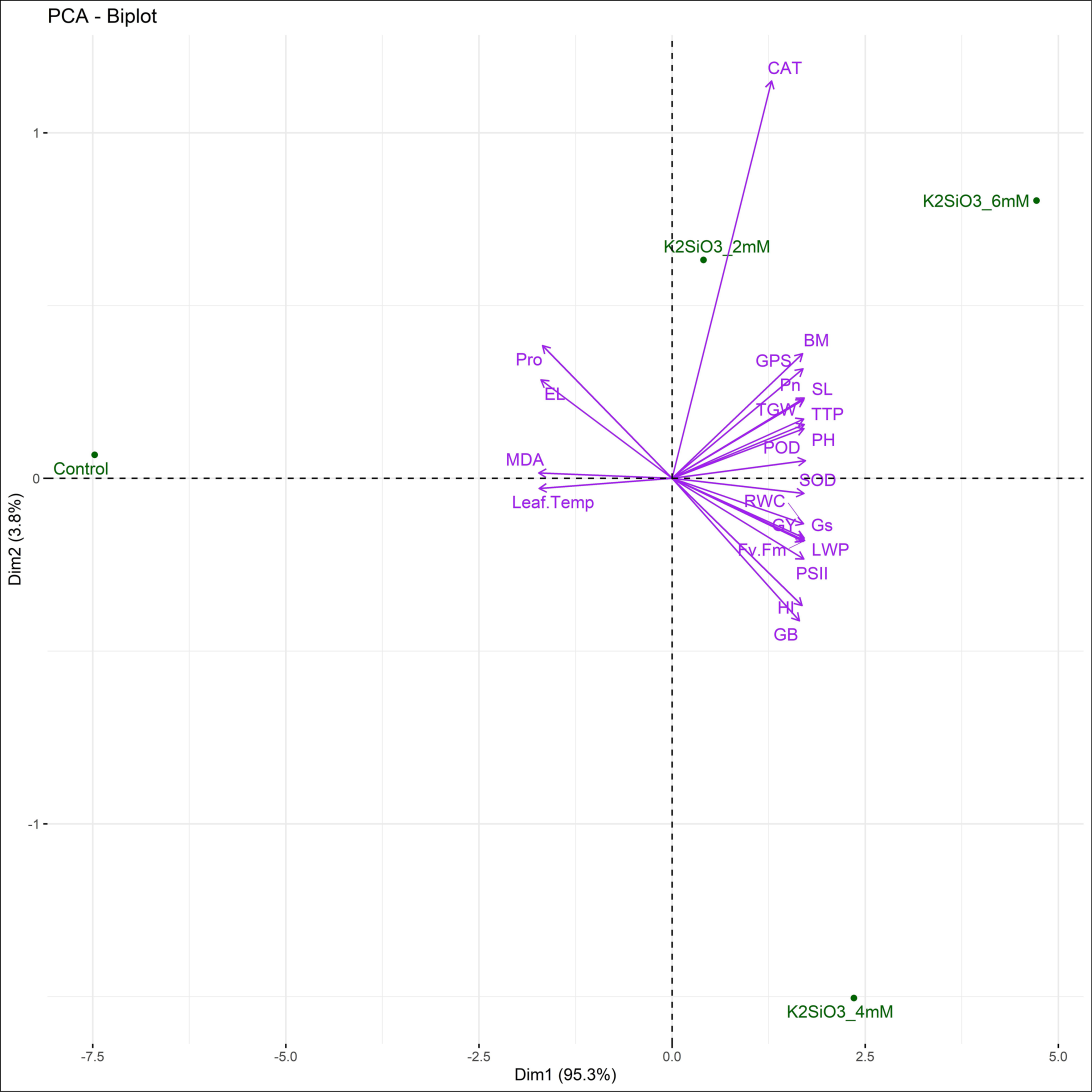
Principal component analysis (PCA) biplot illustrating the relationships between measured traits and potassium silicate (K_2_SiO_3_) treatments under terminal drought stress. Treatments (Control, 2 mM, 4 mM, and 6 mM K_2_SiO_3_) are represented as points, whereas trait vectors indicate their relative contributions to sample separation along the first (PC1) and second (PC2) principal component axes.

**Figure 6:**
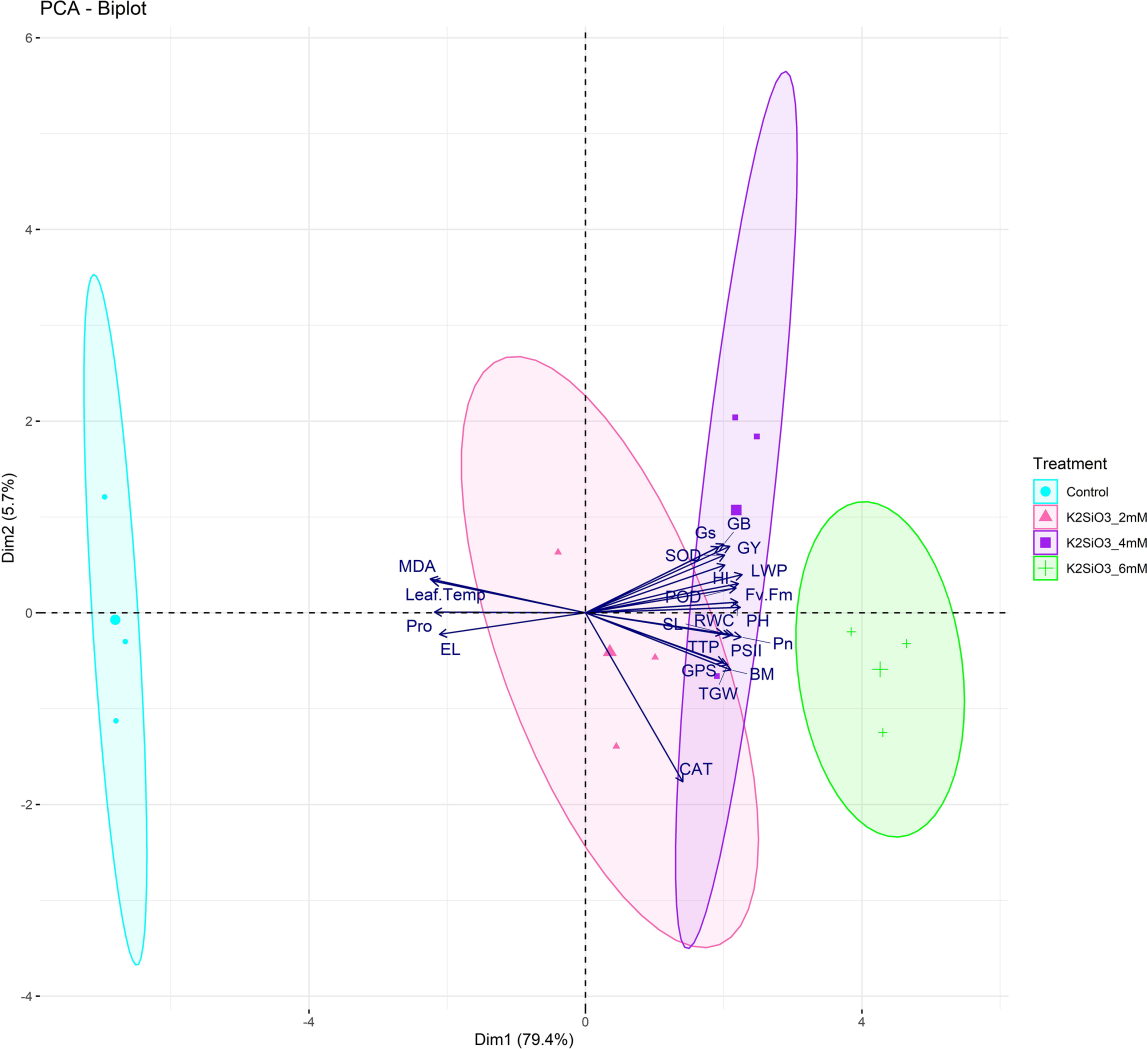
Principal component analysis (PCA) biplot with confidence ellipses depicting the clustering of wheat traits in response to potassium silicate (K_2_SiO_3_) treatments under terminal drought stress. The treatment-wise confidence ellipses show clear separation among groups, with the control treatment clustering with stress-associated indicators such as malondialdehyde (MDA), proline, and electrolyte leakage (EL). In contrast, the 4 mM and 6 mM K_2_SiO_3_ treatments clustered closely with enhanced yield attributes and key physiological and biochemical traits associated with drought tolerance.

Principal component analysis illustrated 95.3% variation on PC1 and 3.6% on PC2, clearly separating treatments (**Figures 5** and **6**). The control clustered with stress indicators (MDA, Proline, EL, Leaf Temp), while K_2_SiO_3_ at 4 mM was strongly associated with yield parameters (GY, TGW, GPS, PH, BM) and photosynthetic traits (Pn, RWC, PSII, Gs, Fv/Fm). The 6 mM treatment clustered with antioxidant traits (SOD, POD, CAT) and osmoprotectants (GB), showing stress resilience (**Figure 5**). PCA ellipses further confirmed treatment separation, with control and treated plants forming distinct groups indicating the varying impacts of K_2_SiO_3_ on treated plants (**Figure 6**).

**Figure 7:**
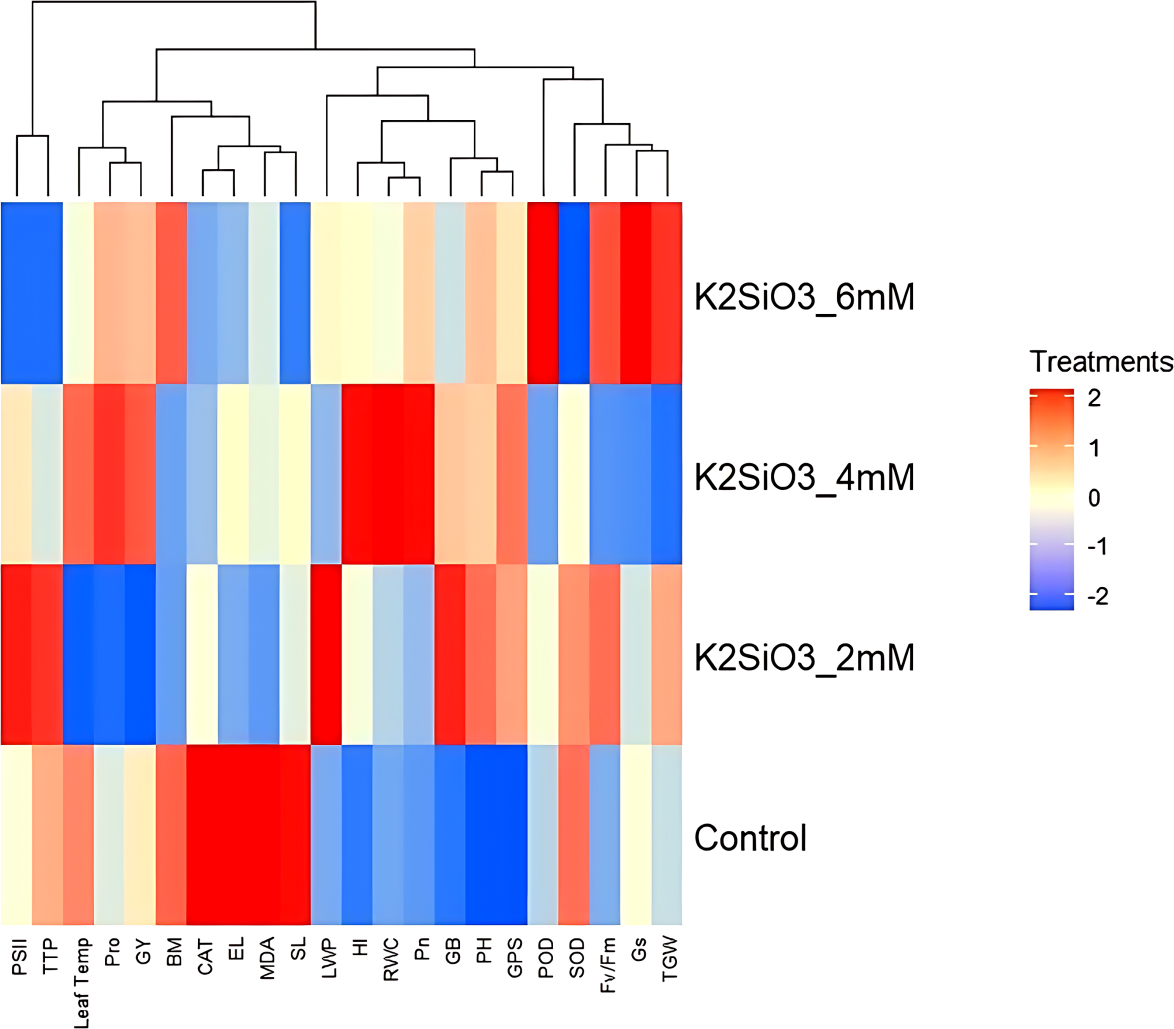
Heatmap illustrating hierarchical clustering of physiological, biochemical, and yield-related traits of the drought-susceptible wheat cultivar ‘Galaxy-2013’ under terminal drought stress across different potassium silicate (K_2_SiO_3_) treatments (Control, 2 mM, 4 mM, and 6 mM). Blue shades represent lower levels of trait expression, whereas red shades indicate higher expression levels. The clustering pattern highlights treatment-specific responses and trait associations under drought conditions.

The heatmap classified treatments into two major groups (**Figure 7**). The control clustered with drought-induced stress parameters (MDA, Proline, EL, Leaf Temp), while 4 mM and 6 mM K_2_SiO_3_ clustered with favorable traits (GY, TGW, RWC, Pn, PSII, Gs, Fv/Fm), confirming that higher K_2_SiO_3_ doses increased drought tolerance in wheat. The 2 mM treatment depicted an intermediate effect, although closer to stress alleviating traits but less effective than 4 mM and 6 mM.

Overall, the application of K_2_SiO_3_ significantly alleviated terminal drought stress in wheat by improving physiological and biochemical tolerance mechanisms in wheat. Among the treatments, both 4 mM and 6 mM concentrations of K_2_SiO_3_ were significantly effective, but 6 mM consistently depicted the highest improvements across physiological, biochemical, and yield parameters.

### 3.5 Gene expression in relation to physiological, biochemical, and yield traits

**Figure 8:**
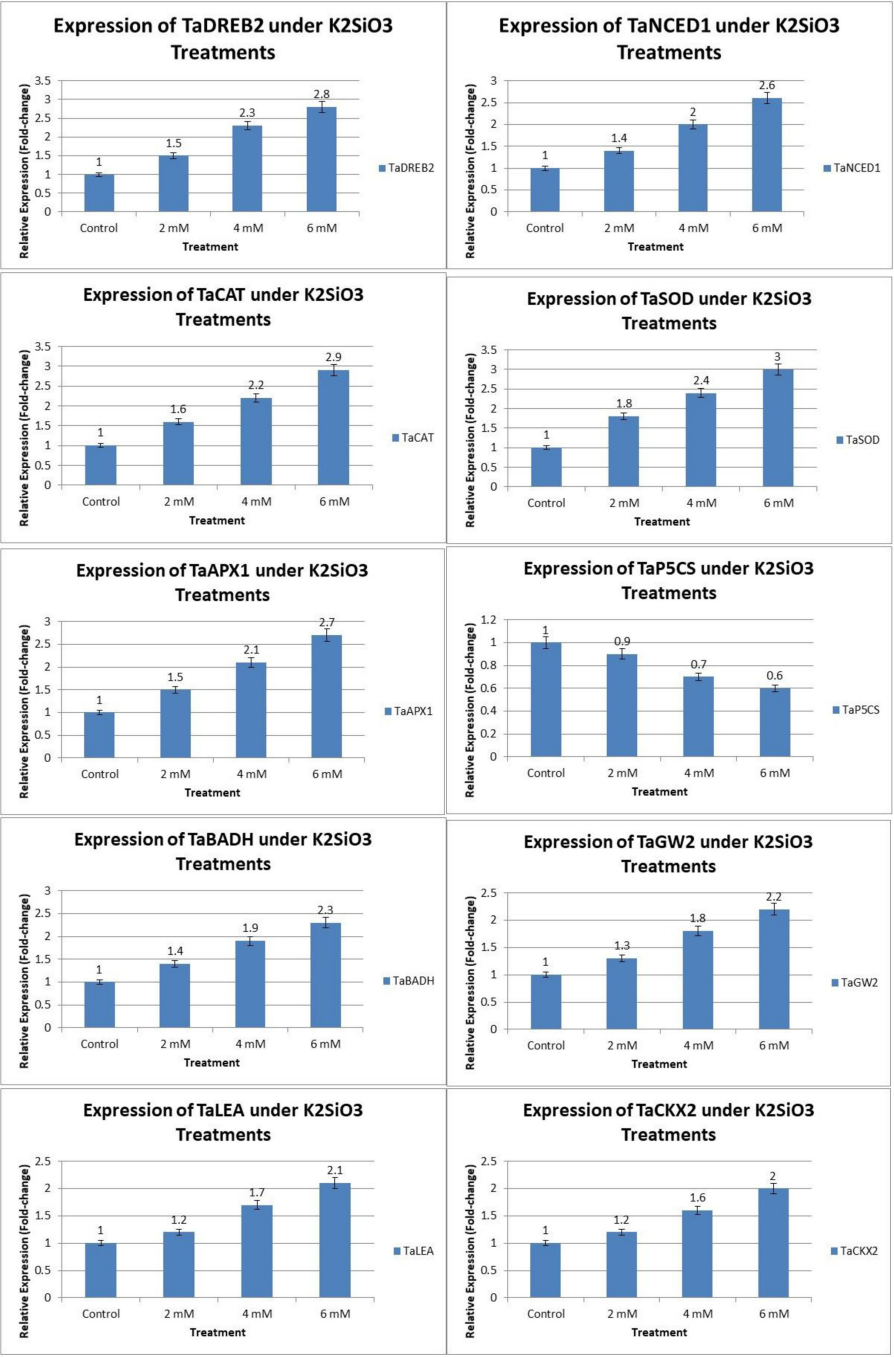
Relative expression profiles of drought-associated genes in the drought-susceptible wheat cultivar ‘Galaxy-2013’ in response to different potassium silicate (K_2_SiO_3_) treatments under terminal drought stress.

The expression of drought-responsive genes in wheat under K_2_SiO_3_ application showed a coherent pattern with the physiological, biochemical, and yield responses observed under terminal drought stress (**Figure 8**). The transcription factor *TaDREB2* exhibited a strong induction, increasing from a relative expression of 1.0 in the control to 2.8-fold at 6 mM K_2_SiO_3_ (**Figure 8**). This upregulation is consistent with improved RWC (79.6% vs. 62.4% in control), higher Pn (21.3 vs. 11.5 µmol CO_2_ m^-2^s^-1^), and enhanced PSII efficiency (Fv/Fm, 0.75 vs. 0.68) (**Figures 1** and **8**). Similarly, *TaNCED1*, a key gene in ABA biosynthesis, was upregulated up to 2.6-fold at 6 mM, which aligns with improved leaf water potential (–1.58 MPa vs. –2.45 MPa in control) and reduced leaf temperature (28.4 °C vs. 33.5 °C) as indicated in **Figure 8**. The antioxidant defense-related genes *TaSOD, TaCAT*, and *TaAPX1* showed the significant increase in expression, with maximum expression levels of 3.0, 2.9, and 2.7-fold, respectively, at 6 mM treatment (**Figure 8**). This enhanced expression corresponds with elevated enzyme activities (SOD: 203 vs. 155 U mg^-1^ protein; CAT: 30.9 vs. 22.4; POD: 21.2 vs. 14.2) and reduced oxidative stress markers, including MDA (5.2 vs. 8.4 nmol g^-1^ FW) and electrolyte leakage (19.6% vs. 35.6%) (**Figures 1**, **2** and **8**). In contrast, the proline biosynthesis gene *TaP5CS* displayed a gradual downregulation (from 1.0 in control to 0.6 at 6 mM) (**Figure 8**), which paralleled the decline in proline content (7.6 → 4.8 µmol g^-1^ FW) (**Figure 2**). On the other hand, *TaBADH*, involved in GB synthesis, showed an increase up to 2.3-fold at 6 mM (**Figure 8**), consistent with the elevated GB content (14.6 vs. 9.2 µmol g^-1^ FW) (**Figure 2**).

The expression of *TaLEA*, encoding late embryogenesis abundant proteins, was also enhanced (2.1-fold at 6 mM) (**Figure 8**) that was correlated with reduced EL (**Figure 1**) and improved membrane stability, Importantly, the yield-associated genes *TaGW2* and *TaCKX2* were significantly upregulated, showing 2.2- and 2.0-fold expression, respectively, at 6 mM (**Figure 8**). Their upregulation aligned with enhanced yield traits, including thousand grain weight (39.8 vs. 33.1 g), grains per spike (49.6 vs. 38.5), grain yield (36.7 vs. 21.3 g plant^-1^), and biomass accumulation (65.8 vs. 52.3 g plant^-1^) (**Figure 3**).

Overall, the results demonstrate that K_2_SiO_3_ application at 6 mM induced a coordinated transcriptional response, where regulatory genes (*TaDREB2, TaNCED1*), antioxidant genes (*TaSOD, TaCAT, TaAPX1*), osmolyte-related genes (*TaP5CS, TaBADH*), protective proteins (*TaLEA*), and yield determinants (*TaGW2, TaCKX2*) regulated synergistically in drought susceptible cultivar ‘Galaxy-2013’.

## 4 Discussion

Drought stress at the terminal growth stage poses a major limitation to wheat productivity in arid and semi-arid regions, largely due to its negative effects on plant physiology, biochemical metabolism, and yield. In present study, the exogenous application K_2_SiO_3_ effectively reduced drought-induced damage in wheat, with the highest benefits reported at the 6 mM treatment. Importantly, among all tested concentrations, 6 mM consistently proved to be the most effective level in improving physiological, biochemical, and yield-related traits under terminal drought stress, indicating an optimal dose-dependent response. The integration of physiological (**Figure 1**), biochemical (**Figure 2**), yield-related (**Figure 3**), correlation (**Figure 4**), PCA (**Figures 5** and **6**), heatmap clustering (**Figure 7**), and gene expression analyses (**Figure 8**) highlights a coordinated drought stress-mitigation mechanism associated with potassium silicate treatment. Since a potassium-only control was not included in the experimental design, the observed responses should be interpreted as effects of potassium silicate application rather than silicon-specific mechanisms.

One of the most striking improvements observed was the enhancement of water relations and photosynthetic efficiency (**Figure 1**). K_2_SiO_3_ treatment significantly increased RWC, Pn, stomatal Gs, and PSII efficiency (Fv/Fm), while simultaneously lowered CT and EL. These improvements were remarkable at 6 mM, where RWC increased to 79.6% compared to 62.4% in control, and Pn rose to 21.3 µmol CO_2_ m^-2^s^-1^ compared to 11.5 in stressed control plants (**Figure 1**). This concentration-dependent improvement suggests that optimal silicon supply enhances water retention and photosynthetic capacity more efficiently than lower doses. Past studies have reported that silicon increases leaf water retention and stabilizes chloroplast ultrastructure, thereby sustaining photosynthesis under drought (Tayyab et al., 2018; Johnson et al., 2022; Dabravolski and Isayenkov, 2024). In the present study, these physiological improvements were observed following potassium silicate treatment, which may contribute to improved plant water relations and photosynthetic stability under drought conditions. The upregulation of *TaDREB2* and *TaNCED1* (**Figure 8**) provides supportive molecular evidence associated with these physiological responses, since these genes regulate stress-inducible pathways and ABA biosynthesis, respectively, thus mediating stomatal regulation and photosynthetic stability under water limited conditions (Wang et al., 2021; Song et al., 2024). This clearly establishes a functional link between physiological responses and molecular regulation under optimal potassium silicate concentration.

At the biochemical level (**Figure 2**), K_2_SiO_3_ significantly induced the antioxidant defense system, as reflected in the upregulation of *TaSOD, TaCAT, and TaAPX1* (**Figure 8**) and the corresponding increase in activities of enzymes SOD, CAT, and POD. These changes were strongly associated with reduced oxidative stress markers, including MDA and EL. A similar role of silicon in boosting antioxidant activity under drought has been widely reported in cereals by Farooq et al. (2014); Ma et al. (2016) and Tripathi et al. (2013). In the current study, the enhancement of antioxidant responses was observed in plants treated with potassium silicate, suggesting that this treatment may contribute to improved ROS detoxification under drought stress. Notably, the highest antioxidant activity at 6 mM further supports the dose-dependent efficiency of potassium silicate in mitigating oxidative damage. The strong positive correlations observed between antioxidant enzymes and yield traits (**Figure 4**) further confirm the key role of ROS detoxification in protecting cellular functions and sustaining productivity. These correlations strengthen the connection between biochemical defense mechanisms and final yield performance. Correlation analysis revealed strong associations among physiological, biochemical, and yield traits, highlighting key indicators linked with improved drought tolerance. These relationships provide deeper insight into the coordinated response of wheat to foliar potassium silicate under terminal drought stress.

Osmolyte metabolism depicted a varying response to K_2_SiO_3_ treatment. While proline content declined under potassium silicate application, consistent with the downregulation of *TaP5CS* (**Figure 8**), GB content increased, corresponding with the upregulation of *TaBADH*. This suggests that K_2_SiO_3_ treatment may have moderated osmotic stress, thereby lowering the need for proline accumulation, while at the same time promoting GB synthesis for membrane and protein stabilization. Similar observations were made by Zhao et al. (2023), who demonstrated that *TaBADH* expression is associated with osmoprotection under water deficit condition. Moreover, the upregulation of *TaLEA* (**Figure 8**) agreed with reduced EL (**Figure 1**) and heatmap clustering (**Figure 7**), endorsing the role of LEA proteins in maintaining cell membrane integrity under dehydration stress. These results indicate that biochemical adjustments are closely coordinated with gene expression changes under optimal silicon treatment. The beneficial effects of K_2_SiO_3_ translated into significant improvements in yield-related traits (**Figure 3**).

Moreover, GY, TGW, GPS and BM illustrated maximum accumulation at 6 mM treatment (**Figure 3**) that was consistent with the higher expression of *TaGW2* and *TaCKX2* (**Figure 8**), which are known regulators of grain size and spike fertility, respectively as reported by Jablonski et al. (2021) and Zhang et al. (2018). Multivariate analyses (**Figures 5**, **6**) and heatmap clustering (**Figure 7**) further confirmed that the 6 mM treatment clustered closely with yield and physiological traits of drought tolerance, while the control was closely spaced with stress markers such as MDA and proline. This clustering pattern further validates that 6 mM is the most effective concentration linking improved physiology, reduced stress indicators, and enhanced yield. These results support recent findings that potassium silicate or silicon-based fertilization practices can enhance assimilate accumulation and grain development under drought conditions (Ahmad et al., 2016; Aurangzaib et al., 2022).

Overall, this study shows that K_2_SiO_3_ treatment was associated with the activation of multiple drought tolerance responses in wheat, integrating molecular, physiological, and yield responses. Importantly, the coordinated improvement across these levels under 6 mM treatment demonstrates a strong functional linkage between gene expression, biochemical regulation, and physiological performance. At the transcriptional level, plants treated with K_2_SiO_3_ showed enhanced expression of drought-responsive regulators *(TaDREB2, TaNCED1*), antioxidant genes (*TaSOD, TaCAT, TaAPX1*), osmolyte regulators (downregulation of *TaP5CS* and upregulation of *TaBADH*), protective proteins (*TaLEA*), and yield-related genes (*TaGW2, TaCKX2*). These changes were directly reflected in physiological resilience (higher RWC, Pn, PSII stability), biochemical stability (enhanced antioxidants, lower ROS damage), and morphological improvements (higher TGW, GPS, and yield). The integrated response across **Figures 1** to **8** confirms that potassium silicate treatment can act as a stress-alleviating and yield-supporting strategy in wheat under terminal drought stress, although the current experimental design does not allow the separation of potassium and silicon-specific contributions. These findings were in complete agreement with recent reports proving silicon fertilization as an effective approach for enhancing cereal resilience to climate-induced stresses (Ahmad et al., 2016; Ashfaq et al., 2025; Zhang et al., 2022; Debona et al., 2017). Overall, key drought-related genes showed varied expression during drought stress under different levels of K_2_SiO_3_. Genes associated with osmotic adjustment (regulating GB and proline accumulation), maintained plant water relations under water-deficit conditions. Antioxidant-related genes reduced oxidative damage under drought stress through triggering the scavenging of reactive oxygen species (ROS). Additionally, stress-signaling and transcription factor genes modulate stress-responsive pathways to initiate various stress responsive mechanisms. Together, the coordinated expression of these genes contributes to enhanced drought tolerance by maintaining physiological stability and agronomic yield, and improving survival under drought conditions in plants treated with potassium silicate under terminal drought stress.

## 5 Conclusion

The expression of drought-related genes in drought susceptible wheat cultivar ‘Galaxy-2013’ was closely associated with the observed physiological, biochemical and growth responses. Upregulation of *TaDREB2* and *TaNCED1* in ‘Galaxy-2013’ due to application of K_2_SiO_3_ corresponded with enhanced drought tolerance through improved stomatal regulation and ABA-mediated stress signaling, which reduced water loss. Moreover, *TaSOD*, *TaCAT*, and *TaAPX1* expression correlated with increased antioxidant enzyme activities, mitigating reactive oxygen species accumulation under stress due to the application of K_2_SiO_3_. In the same way osmolyte-related genes, *TaP5CS* and *TaBADH*, were consistent with proline and GB contents, contributing osmotic adjustment in drought susceptible Galaxy-2013 due to application of K_2_SiO_3_. The upregulation of *TaLEA* due to K_2_SiO_3_ corresponded to enhanced membrane stabilization under dehydration. Similarly, upregulation of grain development-related genes *TaGW2* and *TaCKX2* was associated with improved yield components under water deficit conditions due to K_2_SiO_3_ application. Overall, these molecular responses aligned with enhanced physiological traits such as relative water content, chlorophyll stability, and stress resilience, demonstrating a coordinated gene-to-trait relationship.

Application of K_2_SiO_3_, particularly at 6 mM, effectively alleviated terminal drought stress in drought susceptible cultivar ‘Galaxy-2013’ by improving plant water relations, photosynthesis, antioxidant defense, and yield stability. The observed gene expression patterns provided clear molecular support for these physiological and biochemical improvements. Overall, K_2_SiO_3_ supplementation emerges as a practical strategy to enhance drought tolerance in drought susceptible wheat cultivar and sustained wheat productivity under water-limited conditions.

## Data Availability

The original contributions presented in the study are included in the article/supplementary material. Further inquiries can be directed to the corresponding author.
